# Refractoriness in Sustained Visuo-Manual Control: Is the Refractory Duration Intrinsic or Does It Depend on External System Properties?

**DOI:** 10.1371/journal.pcbi.1002843

**Published:** 2013-01-03

**Authors:** Cornelis van de Kamp, Peter J. Gawthrop, Henrik Gollee, Ian D. Loram

**Affiliations:** 1 Institute for Biomedical Research into Human Movement and Health, Manchester Metropolitan University, Manchester, United Kingdom; 2 School of Engineering, University of Glasgow, Glasgow, United Kingdom; 3 Melbourne School of Engineering, The University of Melbourne, Melbourne, Victoria, Australia; University College London, United Kingdom

## Abstract

Researchers have previously adopted the double stimulus paradigm to study refractoriness in human neuromotor control. Currently, refractoriness, such as the Psychological Refractory Period (PRP) has only been quantified in discrete movement conditions. Whether refractoriness and the associated serial ballistic hypothesis generalises to sustained control tasks has remained open for more than sixty years. Recently, a method of analysis has been presented that quantifies refractoriness in sustained control tasks and discriminates intermittent (serial ballistic) from continuous control. Following our recent demonstration that continuous control of an unstable second order system (i.e. balancing a ‘virtual’ inverted pendulum through a joystick interface) is unnecessary, we ask whether refractoriness of substantial duration (∼200 ms) is evident in sustained visual-manual control of external systems. We ask whether the refractory duration (i) is physiologically intrinsic, (ii) depends upon system properties like the order (0, 1^st^, and 2^nd^) or stability, (iii) depends upon target jump direction (reversal, same direction). Thirteen participants used discrete movements (zero order system) as well as more sustained control activity (1^st^ and 2^nd^ order systems) to track unpredictable step-sequence targets. Results show a substantial refractory duration that depends upon system order (250, 350 and 550 ms for 0, 1^st^ and 2^nd^ order respectively, *n* = 13, *p*<0.05), but not stability. In sustained control refractoriness was only found when the target reverses direction. In the presence of time varying actuators, systems and constraints, we propose that central refractoriness is an appropriate control mechanism for accommodating online optimization delays within the neural circuitry including the more variable processing times of higher order (complex) input-output relations.

## Author Summary

In biology, the control of physiological variables such as body position, blood pressure and body temperature is founded on negative feedback mechanisms governing homeostasic input-output relations. The conceptual models capturing the underlying control principles are often drawn from engineering control theory. The visuo-manual control of external systems (like balancing a stick on the palm of one's hand) has traditionally been interpreted using continuous paradigms such as the servo controller or the continuous optimal controller. These engineering controllers were designed for machine systems with precise sensors, consistent actuators, short time delays and fast computers. Quite the opposite is true for the human movement system that is characterized by long neuromuscular delays, variability, history dependence and fatigue. Serial ballistic control offers an alternative control paradigm in which smooth control proceeds as a sequence of sub-movements each planned using current sensory information but then intermittently executed “open loop”. In the current study we are the first to formally identify refractoriness, a behavioural characteristic that discriminates intermittent (serial ballistic) from continuous control, in the domain of sustained (non-discrete) control of first and second order systems providing definite evidence for the validity of intermittent open-loop control as a paradigm for sustained human control.

## Introduction

Our interactions with the environment include stimuli and responses. The concatenation of successive stimulus-response operations is an ongoing process of which we are often unaware. For example, when manoeuvring a car through heavy traffic we brake and accelerate in response to the other vehicles actions. Usually, the chained actions that we execute during the day occur independently of each other. However, when two unpredictable stimuli are presented closely spaced in time, the response to the first stimulus will, at some point, interfere with the response to the second stimulus [1].

A well-known example of dual-task interference is the Psychological Refractory Period (PRP) effect in human neuromotor control which has been studied extensively using the double stimulus paradigm [e.g. 2]–[6]. The refractory duration is defined as the temporal separation of step-stimuli beyond which there is no interference, that is, the inter-step interval (ISI) up to which the time to respond to the second step (RT2) is elongated relative to the time to respond to the first step (RT1) [6]. The “single channel hypothesis” (as discussed by Smith [7]) provides a possible explanation of this effect and predicts that a decrease in the ISI results in an increase in RT2 by the exact same amount. According to this hypothesis, the intercept of the linear regression function of the elongated RT2 minus the average RT1 without interference should equal the refractory duration.

Most models on stimulus-response operations assume three basic stages of processing: sensory analysis (SA), response planning/selection (RP/S) and response execution (RE). According to the single channel hypothesis some of these processing stages cannot overlap and there is a central bottleneck associated with response selection and response planning [6], [8], [9]. Selecting and planning a response can be done for only one response at a time. Further processing of the second of two closely spaced stimuli is put on hold until the response selection and programming for the first stimulus is complete [10]. Interference between two responses occurs because the first action has already been selected and the second process is completely or partially blocked [11].

**Figure 1:**
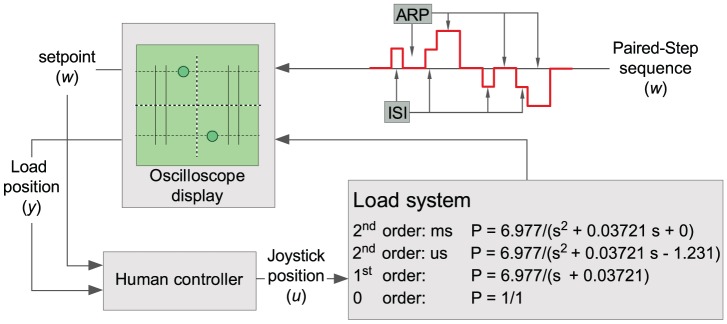
Diagram of the control system and the experimental set up. The system is ‘virtual’ and is controlled through a joystick interface. The participant receives visual feed-back information about the system position through a dot presented on a real oscilloscope. The joystick position defined the system's: position (0 order system), its velocity (1^st^ order system), or its acceleration (2^nd^ order system). While controlling the system, participants were asked to track the position of a second dot displayed on the oscilloscope. The four possible step sequences (uni- and reversed directional step to the left or to the right) of the pursuit target are illustrated by the red line. First and second stimuli are separated by an inter-step interval (ISI), double stimuli are separated by an approximate recovery period (ARP). When applied to a model (as shown in Fig. 7), this sequence is applied as a set-point disturbance.

**Figure 2:**
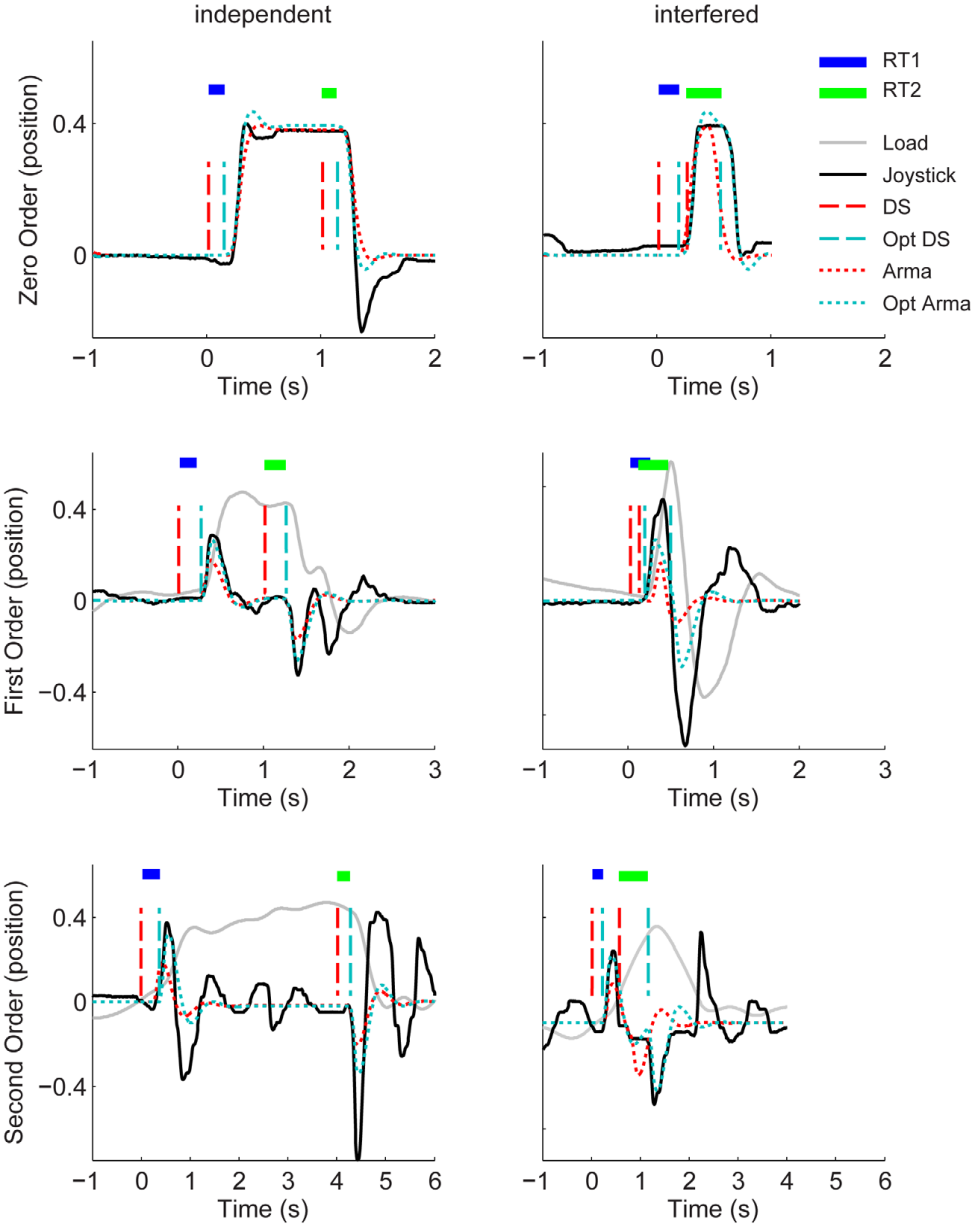
Representative responses; reconstruction of the set-point (stage 1). Panels show representative examples of positional joystick responses over time (blue solid lines) in: Zero Order (top row), First Order (middle row), and Second Order (bottom row) conditions. Left panels show independent responses without interference, right panels show trials with interference between responses to the second and first stimulus. The dashed line (red) shows the time-invariant optimized ARMA fit corresponding to the original/actual double step stimulus (dark blue dashed line). The dotted line (green) shows the best fitting ARMA model corresponding to the non-time-invariant optimised step sequence (dark green dotted line). Estimates of first (RT1 in blue horizontal bar) and second (RT2 in green horizontal bar) delays hover above, and span the interval between the actual and optimised step sequence. System position is displayed by the solid gray line; this is the same as joystick position in the Zero Order case.

In their seminal studies, Craik and Vince [3]–[5] demonstrated the refractory nature of pursuit tracking following an initial response to an unpredicted, discrete step stimulus (cf. Fig. 1). They showed that by decreasing the separation between the onset of the two stimuli (ISI), RT2 was delayed as compared with RT1 and that this delay (according to Vince [5] ranging between 200–500 ms) was most pronounced with short ISIs (typical waveforms of responses that illustrate the occurrence of RT1 and RT2 are presented in Fig. 2). Based on these findings, Craik and Vince argued that human motor control can be described by a servo system, which is operated intermittently rather than continuously and proposed serial, ballistic control at a rate of two to three actions per second [3], [5]. Serial ballistic (or intermittent) control allows for execution of an action followed by observation of the result, before the selection and planning of the next action. This way, smooth control can proceed as a sequence of sub-actions each planned using current sensory information but then executed open-loop (i.e. without being influenced by immediate feedback of the result). Craik argued that this intermittent control (i.e. serial ballistic correction), which was evident in unpredictable discrete movement control, is the actual mechanism even when control was sustained. Under usual circumstances, intermittency is not apparent because participants can predict the required control action and make smooth continuous movements.

Recently, it has been proposed that the PRP effect is naturally interpreted as the open-loop interval associated with an intermittent controller [12], [13]. A small number of authors have advocated that the mechanism of intermittent control (which is salient in discrete responses to step stimuli) may be widely appropriate during sustained movements and postural control [12]–[26]. Using a visuo-manual tracking task in which participants controlled an external unstable second order system whose output was represented by a dot displayed on a real oscilloscope, Loram and colleagues [13] showed that joystick control constrained to be intermittent open loop using gentle taps (in which the thumb or index finger were only in contact with the joystick during the tap) is natural, effective and more robust to unexpected changes than continuous hand contact, works best with a preferred modal rate of about two taps per second, and can explain the upper frequency limit of control by both methods (tapping and continuous contact). According to the authors, serial ballistic (i.e. intermittent) control, at an optimum rate on account of refractoriness, provides a physiologically meaningful paradigm for explaining human neuromotor control [c.f. 13].

The motor control literature circumstantially suggests that the PRP effect is not bound to discrete movements and is apparent over a wider range of stimuli response actions, which have no recognisable beginning and end (e.g. continuous ramp/sine wave tracking [27], articulation of words [28], sports [29], rhythmic movements [30], [31] and handwriting [32]). Refractoriness seems to occur even when the two stimuli are chosen from different sensory modalities, for example, vision and audition [33], and when the first and second response make use of different effector systems, such as one verbal and one manual response [6] and even in joined actions where two operators share common tasks [34]. Given the omnipresence of the PRP effect, refractoriness is considered to be a general phenomenon and served as a textbook example for explaining the various stages of stimulus response processing [6], [10].

One problem is, however, that substantial refractoriness has never been formally identified in the domain of sustained (non-discrete) control actions and whether or not refractoriness generalizes to sustained control is still an open question. This means that, to date, intermittent control in sustained tasks is unproven and disputed [35]. Please note that in the current study, we refer to a low frequency intermittent control process that is clearly different form the high frequency form of clock-driven refractoriness predicted by the Adaptive Model Theory developed by Neilson and colleagues [24], [25] that is characterized by an intermittent interval of 50–100 ms related to pulsatile central neural control at a frequency of 7–10 Hz matching tremor and resonance related discontinuities in human data. Here we wish to study sustained positional control in a reduced but reasonably generic way, under the most precisely controlled experimental conditions, using PRP perspectives from psychology [9] and using engineering control theory as a rigorous interpretational framework [12].

Why has the existence and quantitative value of the PRP effect not been established in sustained manual control of external systems? First, sustained control tasks (e.g. ramp/sine wave tracking, human balance control) lack a clear step in the tracking stimulus. Second, in sustained control it is difficult to determine a clear beginning and end of the response. More generally, distinct bursts of action are difficult to view because muscles and limbs smooth out the transitions between discrete actions giving the impression that we respond continuously instead of intermittently. In other words, due to the dynamics of the (higher order) systems (e.g. the neuro-muscular system, the inertia of an external system, etc.) control features (e.g. the kinematic landmarks indicating the initiation of ballistic control movements) are masked. This has made it challenging to develop a method of analysis suitable to show a possible refractory duration effect in sustained control.

Recently [36], a novel approach to discriminating continuous control from intermittent control and identifying the extent of refractoriness, was developed and tested on theoretical control models and on data of human pursuit tracking (discrete stimuli-response task where control is known to be refractory). As discussed in Section 2.2 of [36], the relation between stimulus and response is modelled as a linear time-invariant (LTI) system together with a varying stimulus delay; an optimization algorithm determines the LTI system together with a stimulus delay for each stimulus which best matches the data. The statistical properties of the estimated stimulus delays are then used to distinguish the competing continuous and intermittent hypotheses and, in the latter case, determine an estimate for the refractory duration (i.e. ISI beyond which there is no interference between stimulus-response pairs). Subsequently the method reveals the relationship between stimulus delay and ISI which enables testing for assumptions like the single channel hypothesis in both discrete and sustained control tasks. In the sequel, this method will be referred to simply as “the method of analysis”.

Here we applied the method of analysis to data collected in participants controlling four different systems. These systems exhibited properties varying in order (0, 1^st^ and 2^nd^) and (for the 2^nd^ order system) in the unstable time constant (marginally stable vs. unstable) representing, in a biomechanical analogy, passive stabilisation. These factors (Order and Stability) make different demands on the human.

Following [23] the “complexity” demand is related to system order and can be expressed as the level of processing that is required to stabilise the system. The level of processing required depends on the number of derivatives involved in mapping the system position to joystick movement in order to stabilise the system. Ongoing stabilisation of a second order system requires processing of system position and system velocity whereas stabilisation of a first order system requires processing of only system position. A zero order system requires no ongoing stabilisation because joystick position imposes no sustained movement (velocity) on the system. The “promptness” demand is related to system stability determined by the unstable time constant [23].

Thus, an experimental distinction is made between discrete and sustained control of movement. Discrete movements like throwing and reaching are ballistic in nature and have a recognizable beginning and end. These characteristics are comparable to controlling a zero order load where the joystick position imposes no sustained movement on the system and no ongoing control is required after tracking the step change in target. Sustained movements like human balance are ongoing (and the system is unstable) which means that sustained feedback is required.

In the current study we test the hypothesis that refractoriness generalizes to sustained control and address the following 3 research questions. Answers to questions 1-and 2 do not require model based assumptions.

1. What is the refractory duration of sustained control (of 1^st^ and 2^nd^ order systems) and does it differ from the refractory duration of discrete pursuit tracking (0 order system)?
2. Is the refractory duration physiologically intrinsic or does it depend on system complexity (determined by the order of the system)?
3. Is sustained visual-manual control serial ballistic?

## Methods

Details with respect to the apparatus, the visuo-manual tracking tasks and the method of analysis have been restricted to the minimum necessary since they are stated more fully in previous work [23], [36], respectively.

### Ethical approval

The experiments reported in this study were approved by the Academic Ethics Committee of the Faculty of Science and Engineering, Manchester Metropolitan University and conform to the Declaration of Helsinki, participants gave written, informed consent to the experiment.

### Procedure, apparatus, and measurement

Thirteen healthy subjects (8 male, 5 female), aged 22–34 years (28±4 years, mean ± S.D.) sat at a table in a self-selected position. Participants used continuous contact of a uniaxial joystick supported on the table surface in front of them to control the left-right position of a dot on a, real, analog oscilloscope placed 50 cm away. This dot represented the position of a one-dimensional (left-right) virtual system (see Fig. 1). Following [23], [36], the virtual systems were constructed using Simulink, were compiled using Real-Time Workshop and executed on a laptop using Real-Time Windows Target within MATLAB v7 (MathWorks) at a sample rate of 1000 samples per second.

In the current study participants controlled four different external systems (c.f. Fig. 1) that exhibited properties varying with respect to the order of the system (0, 1^st^ and 2^nd^) and (for the 2^nd^ order system) the unstable time constant of the system (marginally stable vs. unstable) that, in a biomechanical analogy, represented passive stabilisation. All these systems have been used in previous experiments (0 order: [36], 1^st^ and 2^nd^ order (marginally stable and unstable): [23] (load 5, 1 and 2, Table 1)). The second order stable system can be thought of as simply being a ‘mass’ with no destabilising effect from gravity, whereas the unstable system has a time constant of 0.92 s equivalent to that experienced by an adult during normal standing [c.f. 23]. In the second order conditions, the position of the joystick modulated the acceleration of the system. For the first order condition we removed the mass content of the system and now the position of the joystick specified the velocity of the system. For the zero order condition the position of the joystick specifies the position of the system.

**Table 1:** Selected ISIs.

System Order	ISI (s)							
zero	0.05	0.10	0.15	0.20	0.25	0.30	0.50	1.00
first	0.10	0.15	0.20	0.25	0.35	0.50	1.00	2.50
second	0.15	0.25	0.35	0.45	0.55	0.65	1.50	4.00

The eight selected ISIs for the three different system orders.

Our set up and instructions were designed to elicit the most continuous behaviour possible. The trial order was randomized to eliminate learning effects. All participants were familiarized with the control tasks. Participants were informed that for zero order systems the position of the joystick instantly modulated the position of the system whereas for the first and second order system the position of the joystick modulated, respectively, the velocity and acceleration of the system. After some practice, all participants were able to control the second order system within the limits imposed by the oscilloscope display. The purpose of the explanation/familiarization was to overcome the initial (steepest) part of the learning curve. In the unusual event that the participant failed to keep the position of the dot within the oscilloscope's display limit, the system was swiftly returned to the centre position and its velocity and acceleration were reset to zero. Three participants were particularly gifted in controlling the external systems because of extensive previous experience and/or a gaming background. Seven had only moderate previous experience of this task.

Just above (1 cm) the dot representing the external system a second dot, representing the target position, was displayed on the oscilloscope (see: Fig. 1). To minimize the degree to which participants could anticipate their pursuit tracking responses we designed the following tracking target step sequence. Participants were told that every now and then, the target would jump to the left or to the right. The only instruction given to the participants was to respond as quickly and accurately as possible to each step in target position and that the deviation between target position and system position (i.e. the top and bottom dots on the oscilloscope) was the measure of performance. Participants were not informed about the amplitude or direction of these jumps. Spatial unpredictability of the double step stimuli was achieved by varying the direction of the step in target position (left-right, right-left, left-left-right to centre, right-right-left to centre, see Fig. 1). Temporally, stimulus predictability was eliminated by varying the ISI (see Table 1). The eight different double and/or triple step stimuli were presented four times in a randomized order. The time it takes a participant to recover from a step response increases with the order of the controlled system [c.f. 27]. Based on pilot data it was estimated what ISI would be sufficient to recover from a step response when controlling a zero, first, and second order system. The last two ISIs were chosen well beyond this ‘Approximate Recovery Period’ (ARP) to serve as an independent base measure. The remaining six ISI (<ARP) were chosen such that they would span the hypothesised refractory duration for that specific condition (see Table 1). The ARP after a double and/or triple step stimulus (see Fig. 1) was randomly chosen within a one second range including the (maximum) ARP specific for each system order (i.e. 0: 1–2 s. 1^st^: 2–3 s. 2^nd^: 4–5 s.). The trial duration was determined by the sum of the selected ISI and ARP attributed to the specific system order condition. Since participants were encouraged to perform at their best, a break of up to five minutes was offered between trials.

### Method of analysis

The method of analysis proceeds in three stages [c.f. 36]. The first two stages do not require model based assumptions and quantify refractoriness, the key feature discriminating serial ballistic (intermittent) from continuous control.

#### Stage 1: Reconstruction of the set-point

Following [36], we estimated the step-joystick time delay for each first and second step (i.e. RT1 and RT2) by modelling the closed loop relationship between the target (step sequence) signal and the joystick position as a low order, zero delay, autoregressive moving average (ARMA) process. The ARMA model's order (10th) was set such that while the number of coefficients was sufficient to capture the key features of the participants' responses (see Fig. 2) they never exceeded Akaike's Information Criterion [c.f. 36], [37]. Next, we reconstructed the step sequence by sequentially and individually adjusting the instant of each step to optimise the fit of the ARMA model. This was done in a time-invariant and in a non time-invariant way. Time-invariant optimization means that a best ARMA fit is achieved by reconstructing the step sequence using equal adjustments of the instant of all steps (basically determining the time delay of the ARMA model). The non-time-invariant optimization method allowed different adjustments of the instant of the first and second steps and can be referred to as a ‘set-point reconstructed ARMA model’. If the description can be improved by optimising the delay to each step, this procedure will provide a distribution of delayed responses to each first and second step (RT1 and RT2). Analysis of these delays with respect to ISI can test for refractoriness [c.f. 36].

#### Stage 2: Statistical analysis of RT1s and RT2s

Following the data analysis [36], the main measures of interest were the distributions of RT1 and RT2. Unless stated otherwise, individual values are quoted as mean (± standard deviation) and a repeated measures ANOVA design is used to test for the effects of Step Number (first and second ), System Order (0 order, 1^st^ order, 2^nd^ order), ISI (level 1 through 8), and their interactions. As recommended in [38], the average of the Greenhouse–Geisser and Huyhn-Feldt corrections of degrees of freedom was used based upon the estimates of sphericity. Post-hoc ANOVAs were run to evaluate significant main and interaction effects. By design, the data set can be subdivided into a group of reversed step-pairs and a group of unidirectional step-pairs which we analyzed separately.

Without requiring any model based assumptions, the following tests provide evidence which can discriminate against continuous control and quantify the extent of refractoriness in this task [36].

- Are the ranges of RT1 and RT2 equal? A hypothesis of zero refractoriness would predict equal ranges (5–95^th^ percentile) in distributions of RT1 and RT2.
- Is RT2 greater than RT1? A hypothesis of zero refractoriness would predict equal delays.
- Is there an interaction between the factors: Step Number (first and second) and ISI (levels 1 through 8)? A hypothesis of refractoriness would predict an interaction between ISI and Step Number. Refractoriness would increase RT2 but not alter RT1.
- Is RT1 independent of ISI? A refractory hypothesis would predict that RT1 is independent of ISI.
- What is the ISI up to which RT2 is significantly greater than RT1? Testing within each level of ISI for differences between RT1 and RT2 will reveal the ISI up to which there is interference between RT2 and RT1 and quantifies the duration of refractoriness.
- What is the maximum increase in RT2? Using linear regression to fit RT2 v ISI for ISIs where RT2 is significantly greater than RT1, the extent of refractoriness was quantified using the regression intercept (ISI = 0) minus average RT1.

#### Stage 3: Model based interpretation of delays

- What is the open-loop interval using the single channel/IC model hypothesis [12]? A single channel hypothesis of intermittent control in which a response is triggered by the first step predicts a slope of −1 in the relationship between average RT2 and ISI for ISI<open-loop interval [6]. The above regression method was repeated assuming a least mean squares fit with slope constrained to −1.

## Results

With only a single familiarisation session of less than 10 minutes, all subjects were able to control the second order system while tracking the step sequence within the limits of the oscilloscope screen for the duration of one trial (∼200 s).

### Representative pursuit tracking responses to double step stimuli when controlling zero, first, and second order systems

Fig. 2 illustrates the comparison between responses without interference and responses that show evidence of interference. With long ISIs (left panels, Fig. 2), responses to the second steps are similar to responses to the first steps. Looking at the examples for which the ISIs were small (right panels, Fig. 2) we see that responses to the second steps are interfered by responses to the first step. This interference is characterized by an elongation of the second response relative to the first response (in Fig. 2. compare the blue (RT1) horizontal bar to the green (RT2) horizontal bar). With large ISIs RT1s are comparable to RT2s. With small ISIs, however, RT2s are clearly longer than the RT1s.

### Reconstruction of the set-point (stage 1): representative results

Overlaying in Fig. 2: i) a participant's typical response in solid black, ii) the ARMA prediction in dotted red and iii) the set-point reconstructed ARMA prediction in dotted cyan shows (as one would expect) no real difference for the independent responses (c.f. left panels Fig. 2). When focusing on the responses hypothesised to be vulnerable to interference (Fig. 2 right panels) we found that reconstructing the set-points resulted in a better (ARMA) description of the data (this is evident when we look at the response amplitude and even more so when we look at the actual timing of the responses). The blue and green bars displayed above and spanning the interval between the actual and optimised steps displayed in Fig. 2 exemplify the delays identified in stage 1 of the method of analysis. Whereas RT1 (blue) seems to equal RT2 (green) in the independent responses (left panel Fig. 2.) refractoriness is quantified by the elongation of RT2 in the responses subject to interference (right panel Fig. 2).

### Statistical analysis (stage 2): group results

**Figure 3:**
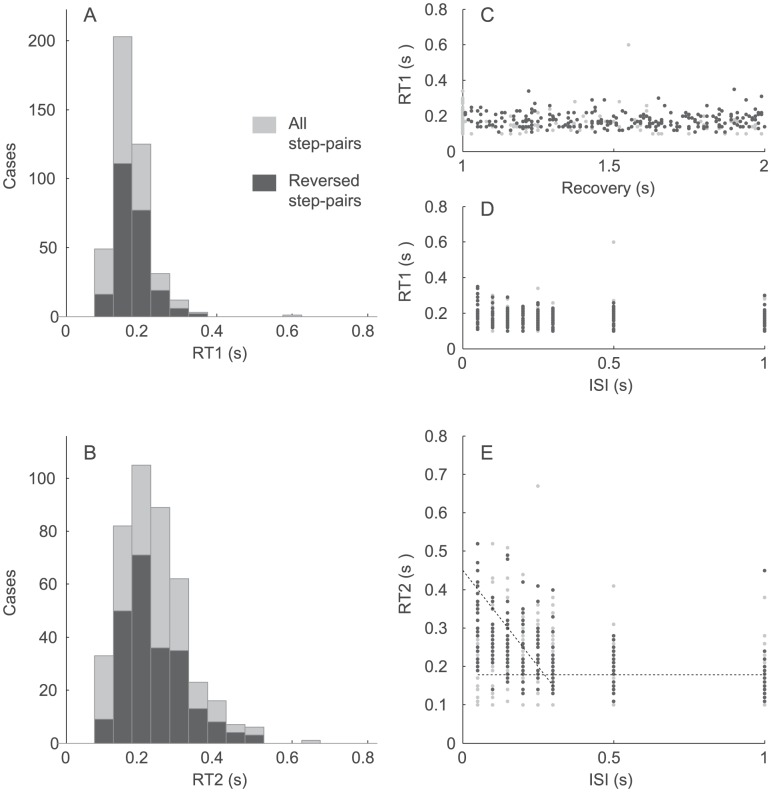
Group results: Distributions. The system condition is Zero Order. Panels A and B show the distribution of RT1 (panel A) and second RT2 (panel B) for all step-pairs (light) and reversed step-pairs only (dark). Panels C and D show individual values of RT1 as a function of the Recovery Period (panel C), and the inter-step interval (ISI, panel D). Panel E shows individual values of RT2 against ISI.

The distributions of RT1 and RT2, including range and central values are clearly different (Fig. 3 A, B). Whereas RT1 is independent of ISIs and recovery period (Fig. 3 C, D), RT2 shows increased range and central value at lower ISIs (Fig. 3E).

**Figure 4:**
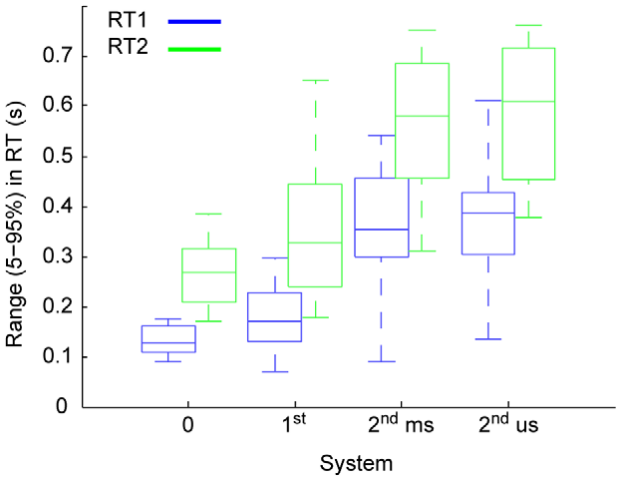
Group results: Ranges. The inter-participant (13 in each system condition) ranges (5–95%) in RT1 (blue) and RT2 (green). Each box shows, the median range (central mark), the 25th and 75th percentile range (the edges of the box are), and the most extreme data points not considered outliers (the whiskers) of these ranges. The maximum whisker length is 1.5. Data points are drawn as outliers ‘+’ if they are larger than q3 + w(q3 - q1) or smaller than q1 - w(q3 -q1), where w is the whisker length and q1 and q3 are the 25th and 75th percentiles, respectively.

The range in RT was systematically affected by Step Number and System Order (Fig. 4).

Combining all step-pairs directions (i.e. reverse and same) and all system orders, the mean range in RT was significantly higher for step 2 than for step 1 (443±185 ms, 260±148 ms, F(1, 12) = 102, p<.0001). The mean range in RT increased significantly through zero, first, and second order stable and second order unstable systems (202±85 ms, 263±137 ms, 462±171, 479±182 ms respectively, F(3, 36) = 53.9, p<.0001). We found no interaction effect between any of the experimental factors: Step Number (first and second), System Order (0 order, 1^st^ order, and 2nd order), and ISI (level 1 through 8).

Separate tests for step-pairs in the reversed or same direction revealed the same main effect for Step Number and System Order on mean range in RT (as per Fig. 4).

**Figure 5:**
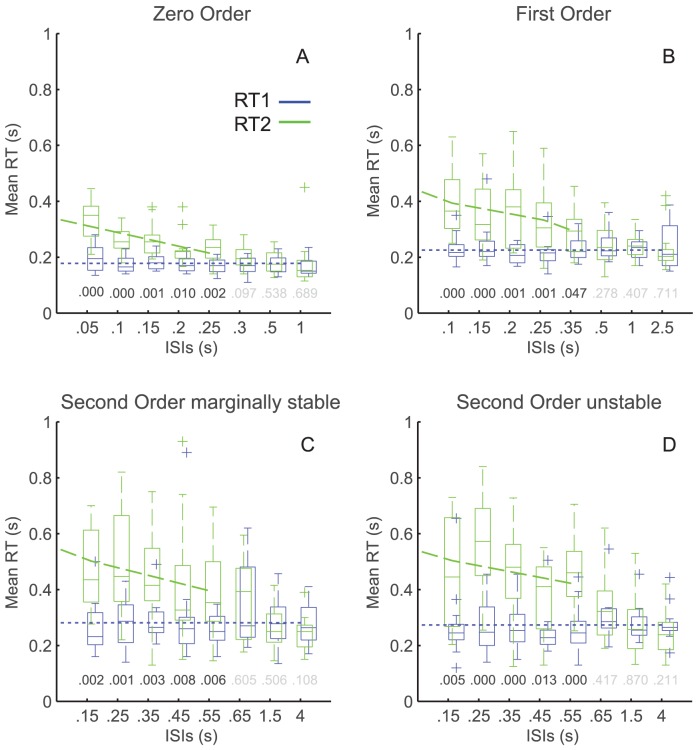
Group results: statistical analysis of Mean delays (stage 2). The four panels: Zero Order System (A), First Order System (B), Second Order marginally stable System (C), Second Order unstable System (D), show the inter participant mean RT1 (blue) and RT2 (green) against ISI for the reversed step-pairs stimuli only (for details regarding the box plot’s constituents see caption Fig. 4). The P-values of the ANOVA’s post hoc test are display above each ISI level (black if significant, gray if not). The blue dotted line shows the mean RT1, the dashed green line shows the unconstrained regression linear fit between (interfered) RT2 and ISIs.

RTs showed significant, substantial refractoriness for all system orders, but in the sustained control conditions (1^st^ and 2^nd^ order) only for reversed step-pairs stimuli (Fig. 5).

#### Zero order system

Reversed step-pairs: Combining all ISI levels, the mean RT was significantly higher for step 2 than step 1 (233±74 ms, 176±35 ms, F(1, 12) = 24.1, p<.0005, Fig. 5 A). Combining RT1s and RT2s showed a significant increase in RT with decreasing ISIs (265±92 ms, 217±56 ms, 221±60 ms, 201±53 ms, 197±50 ms, 185±38 ms, 178±36 ms, 172±50 ms, F(7, 84) = 11.2, p<.0001). The significant interaction effect between Step Number and ISI, (F(7, 84) = 8.96, p<.0001) indicates that reducing the ISI had different effects on RT1 compared to RT2. Conducting two separate post-hoc tests to break down the interaction, showed a significant effect of ISI on the RT2s, (F(7, 84) = 13.4, p<.0001), but not on the RT1s.

We quantified refractoriness in two ways. RT2 was delayed relative to RT1 for ISIs up to 250 ms (paired comparison of Step Number at each ISI: for p-values see Fig. 5, A). The maximum increase in RT2 was 157 ms (subtracting the average RT1 (176 ms) from the intercept (334 ms) of the regression line of mean interfered RT2s over ISIs (c.f. Fig. 5, A).

Unidirectional step-pairs: analysis of the unidirectional cases showed a significant main effect of Step Number (250±75 ms, 177±45 ms, F(1, 12) = 10.1, p<.01). In contrast to the reversed cases, we did not find a significant difference between ISIs (210±77 ms, 211±73 ms, 230±72 ms, 216±63 ms, 225±112 ms, 214±51 ms, 206±60). The significant interaction effect (F(7, 84) = 2.66, p<.05) however indicates that reducing the ISI had significant effects on RT2 (F(7, 84) = 12.4, p<.005), but not on the RT1s.

RT2 was delayed relative to RT1 for ISIs up to 300 ms and the maximum increase in RT2 was 78 ms (subtracting the average RT1 (177 ms) from the intercept (255 ms).

#### First order system

Reversed step-pairs: over all ISIs, the mean RT2 was significantly higher than mean RT1 (308±149 ms, 230±56 ms, F(1, 12) = 19.7, p<.0005, Fig. 5 B). Combining RT1s and RT2s showed a significant increase in RT with decreasing ISIs (315±125 ms, 298±115 ms, 294±131 ms, 270±108 ms, 263±70 ms, 248±66 ms, 235±41 ms, 235±80 ms, F(7, 84) = 3.1, p<.05). The significant interaction effect (F(7, 84) = 10.6, p<0.0001) shows that reducing the ISI had significant effects on RT2 (F(7, 84) = 6.82, p<.0005), but not on the RT1s.

RT2 was delayed relative to RT1 for ISIs up to 350 ms and the maximum increase in RT2 was 204 ms (subtracting the average RT1 (230 ms) from the intercept (434 ms).

Unidirectional step-pairs: analysis of the unidirectional cases showed no significant main or interaction effects and in contrast to the reversed step-pairs (and to the results in the 0 order system condition) there was no evidence for refractoriness.

#### Second order system, marginally stable

 Reversed step-pairs: the mean RT2 was significantly higher than mean RT1(386±178 ms, 287±110 ms, F(1, 12) = 17.9, p<.005, Fig. 5 C) over all ISIs. Combining RT1s and RT2s showed a significant increase in RT with decreasing ISIs (370±162 ms, 399±192 ms, 370±160 ms, 358±206 ms, 320±139 ms, 348±140 ms, 272±89 ms, 258±72 ms, F(7, 84) = 3.1, p<.05). The significant interaction effect (F(7, 84) = 5.68, p<.005) shows that reducing the ISI had significant effects on RT2 (F(7, 84) = 6.1, p<.01), but not on the RT1s.

RT2 was delayed relative to RT1 for ISIs up to 550 ms and the maximum increase in RT2 was 256 ms (subtracting the average RT1 (287 ms) from the intercept (543 ms).

Unidirectional step-pairs: analysis of the unidirectional cases showed no significant main or interaction effects and in contrast to the reversed step-pairs (and to the results in the 0 order system condition) there was no evidence for refractoriness.

#### Second order system, unstable

Reversed step-pairs: Combining all ISI levels, the mean RT2 was significantly higher than mean RT1 (395±176 ms, 272±88 ms, F(1, 12) = 19.5, p<.001, Fig. 5 D). Combining RT1s and RT2s showed a significant difference between ISIs (362±192 ms, 422±205 ms, 358±164 ms, 305±127 ms, 360±148 ms, 321±115 ms, 275±91 ms, 263±76 ms, F(7, 84) = 3.9, p<.01). The significant interaction effect (F(7, 84) = 5.9, p<.005) shows that reducing the ISI had significant effects on RT2 (F(7, 84) = 5.82, p<.001), but not on the RT1s.

RT2 was delayed relative to RT1 for ISIs up to 550 ms and the maximum increase in RT2 was 263 ms (subtracting the average RT1 (272 ms) from the intercept (535 ms).

Unidirectional step-pairs: analysis of the unidirectional cases showed no significant main or interaction effects and in contrast to the reversed step-pairs (and to the results in the 0 order system condition) there was no evidence for refractoriness. Combining all second order cases (stable and unstable) to improve power produced the same results.

### Single Channel interpretation of RT interference (stage 3)

**Figure 6:**
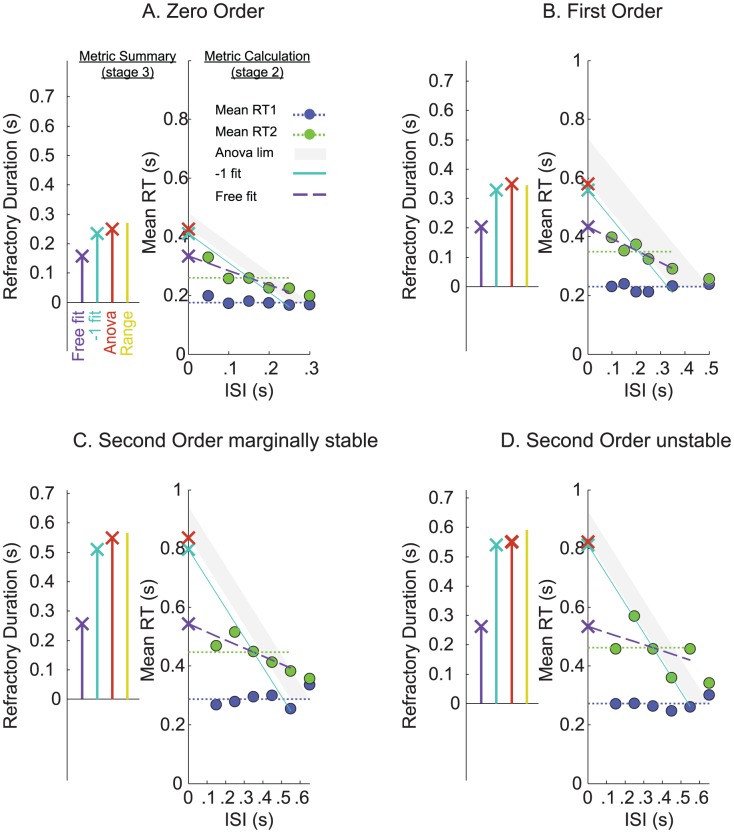
Estimates of the refractory duration. The four panels: Zero Order System (A), First Order System (B), Second Order marginally stable System (C), Second Order unstable System (D), show -in the right axes- how each metric is calculated. Plotted as a function of ISI are mean RT1 (blue circles) and mean RT2 (green circles). The gray area describes the lower and upper limits of the ANOVAs ‘general linear model’ significance, the cyan solid line shows the least mean squares fit between RT2 dependent upon ISI with slope constrained to −1, the magenta dashed line shows the unconstrained regression linear fit between RT2 dependent upon ISI, crosses displayed on the y-axes show the intercepts of these function, and the dotted blue line shows the mean RT1 which served as a baseline for the left axes that summarizes the four estimates of the refractory duration: the intercept of the unconstrained regression fit (magenta), the intercept of the −1 regression fit (cyan), the ANOVA metric (brown), and the Range in RT2 (yellow).

Fig. 6 shows that in all four conditions, the Single Channel (or intermittent Control model) interpretations of RT interference (i.e. the linear interpolation of the ISI up to which RT2 was significantly greater than RT1 (as demonstrated by the ANOVA) and the set slope (−1) regression line through delayed RT2) were closely related and corresponded to the average range in RT2. These predictions of the refractory durations (c.f. Fig. 6) overestimated the refractory duration predicted by the intercept of the unconstrained linear regression through interfered RT2.

All estimates of the refractory duration increased with System Order and were independent of system stability.

The average RT1 (i.e. the baseline of the refractory duration) also increased with System Order and was independent of system stability.

## Discussion

### Summary of results

In this study, for visual-manual control, we formally identified refractoriness, the key feature discriminating serial ballistic (intermittent) from continuous control, in the domain of both discrete (0 order systems) and sustained (1^st^ and 2^nd^ order systems) control actions. Our results showed that delays to the second step were on average longer than delays to the first step. This finding leads to the rejection of a hypothesis of zero refractoriness that predicts equal ranges and equal averages in RT. Our results showed the interaction between Step Number and ISI that was predicted by the alternative hypothesis of refractoriness. Breaking down this interaction showed that whereas delays to the first step were independent of ISI, delays to the second step increased with decreasing ISI levels. The ISI level up to which there was interference between RT1 and RT2 provided an upper limit estimate of the refractory duration which depended upon system order (250, 350, 550 ms for 0, 1^st^, and 2^nd^ order respectively, n = 13, p<0.05) but were independent of system stability. A lower limit estimate of the refractory duration was provided by the maximum increase in RT2 (∼150, ∼200, and ∼250 ms; quantified using the unconstrained regression fit intercept, c.f. Fig. 6). In sustained control (1^st^ and 2^nd^ order systems), refractoriness was only identified when the target reverses direction. The main issues for discussion are: Significance for the continuous versus intermittent control debate, Rationale for serial ballistic (or intermittent) control of human movement, Difference between marginally stable and unstable second order systems, Difference between unidirectional and reversed direction results, Interpretation of our evidence for refractoriness within the intermittent control framework, Why is the refractory duration so long?, and Applicability to other tasks.

### Significance for the continuous versus intermittent control debate

The traditional paradigm for modelling negative feedback control is the servo controller or the continuous optimal controller [39], [40], [41]. Recently, experimental evidence has been presented [35] in which the authors advocate that postural responses to external stimuli are dominated by continuous feedback and cannot be explained by intermittent control. Although continuous control is currently the dominant paradigm, circumstantial evidence for intermittency in human motor control has been observed repeatedly and this issue is currently regarded as an unsolved open question (for overview c.f. [42]). With no modelling assumption this current study provides evidence of refractoriness in sustained visuo-manual control. A continuous (LTI) model cannot reproduce this data and therefore a wider, non LTI, paradigm is required for interpreting visual-manual control [42].

Refractoriness is associated with response selection and response planning [c.f. 7]. The single channel explanation of dual-task interference stresses the psychological relevance of these processes in human movement control [2], [3], [6]. From a control engineering perspective this mechanism is naturally interpreted within an intermittent control framework [12]–[26]. So is there a rationale for serial ballistic or intermittent open loop predictive control based on rigorous engineering principles and relevant to human control?

### Rationale for serial ballistic (or intermittent) control in man and machine

If a system has a pure time delay, the appropriate engineering solution is a predictor [14]–[18], [43]. A predictor [44] is a feed-forward element that, based on an internal system model, can eliminate the time-delay from the feedback loop. As discussed in [43], several approaches for reducing controller design and performance analysis to the delay free case, also applicable to the control of unstable systems such as the human balance system [45]–[48], have been applied in an number of situations including in the engineering literature [39], [49], [50] and in the physiological literature [51], [52]. A predictor requires that the system is consistent, known, and therefore, predictable.

Intermittent control is the appropriate engineering solution to control problems in which there is a time consuming online computational process [12], [43], [53].

When the actuators and the system being controlled do not change with time, and there are no constraints, then controllers can use parameters which are computed offline, such as the gains of a simple or optimal continuous feedback controller. In such cases the control signal can be computed rapidly from measured quantities and the reference signal. However, when the actuators or system change with time, or there are constraints, then online optimization and computation of the control signal is desirable. Intermittent open loop predictive control uses an intermittently moving time horizon which allows slow optimization to occur concurrently with a fast control action. This approach allows handling of time varying systems and constraints at the expense of increased online computational requirement [53]. Thus, intermittent control provides for a time consuming online optimization process which lies at the heart of flexible predictive control.

As stated above, a predictor requires that the system, actuator, and constraints are consistent, and therefore, predictable. However, for neuromuscular control systems consistency is the exception rather than the norm. First consider the time delays. In human motor control, delays include neural transmission and varying degrees of sensori-motor processing according the neural pathways involved. In lower order, peripheral, control processes such as reflex mechanisms maintaining a joint angle in which flexibility is limited to changing the gain and threshold of the feedback, time delays are rather small (40–100 ms), with low temporal jitter. In higher order, central, control processes which allow more flexibility, including choice over direction and timing of response, delays are both larger (>120 ms) and more variable and are restricted to a low frequency bandwidth [13], [23]. Second, the actuator system (muscular), sensory, and processing system (neural) intrinsic to biological control are inherently noisy, variable and subject to signal dependent noise, fatigue, and time varying properties such as thixotropy in the case of muscles [54]–[57]. Third, the constraints determined by environmental factors, changing goals and priorities, and neuro-muscular biomechanical limits can vary considerably with time and even fixed (biomechanical) constraints defy simple, algebraic, pre-computed solutions [53]. The inherent flexibility and predictive nature of higher order, central control mechanisms seems suited to the intermittent, open loop predictive control paradigm. In agreement with psychology, refractoriness seems appropriate for a control mechanism that makes choices and intermittently inhibits alternative control actions [11] to facilitate appropriate response selection and response planning.

Recently, Loram and colleagues [13] provided evidence that control which is explicitly intermittent is particularly robust and effective in controlling a system that is changing unpredictably (different joystick gains). A possible explanation of that effectiveness is that if the system is open loop, causality between input and output can be identified more clearly. Even though a simple inverted pendulum like system can be controlled using continuous linear feedback, an intermittent control structure allows greater flexibility and usefulness while still being effective.

Within the intermittent control scheme, predictive computation is most efficient when the intermittent open-loop interval is greater than the system's total time delay [43]. This provides an expectation that the intermittent interval will increases as the feedback time delay increases. As the order of the controlled system increases, the feedback time delay also increases in association with the increased difficulty of predicting the evolution of states and the increased number of choices of control [23]. Thus we expect time delay and intermittent interval to increase together as system order increases from zero to second. That prediction is confirmed by the results of this experiment.

### Difference between marginally stable and unstable second order systems

We found no difference in results between the marginally stable and unstable system conditions. This is in line with findings by Loram et al. [23] where the primary difference in cognitive demand (as measured by the feedback time delay) was between first and second order systems and not between second order systems of different stability.

One solution to an increase in system instability would be to reduce the feedback time delay (effectively increasing the control bandwidth). Loram and colleagues [23] demonstrated that participants were, however, unable to reduce their delay. This was interpreted as a processing constraint imposed by the order of the system. While stability alters the required promptness of response and affects control performance measured by system position variance, system order increases the cognitive demand by increasing the number of variables (e.g. system position and velocity rather than just position) that the controller has to process in order to stabilise the system. Thus, the demands of system stability and system order are different.

The requirement for more flexible, intentional control mechanisms is one possible justification for central refractoriness. Central refractoriness is naturally expressed as an intermittent control mechanism [12] and intermittent control is an appropriate control mechanism for accommodating time varying systems, actuators, and constraints, including the more variable processing times of higher order (complex) input-output relations [53].

### Difference between unidirectional and reversed direction results

In sustained control, refractoriness was only identified when the target reversed direction. In principle it is possible that our method of analysis is less sensitive to features in the unidirectional responses compared to the reversed responses and that the method's sensitivity declines with higher order systems because the control signal is more variable. Thus, we cannot eliminate the possibility that participants were also refractory during sustained control in the unidirectional cases and that our method of analysis did not detect this refractoriness. Since our method has been validated using model simulation data with varying levels of noise [c.f. 36], we consider it more likely that in sustained control (1^st^ and 2^nd^ order systems) refractoriness does not occur in unidirectional responses.

This finding is in line with previous experimental work [58] showing that stopping ongoing action is subject to refractoriness while responses to stimuli to continue an ongoing action do not produce a refractory duration effect. Together, these suggest that during sustained control, unidirectional responses are online adjustments to the original plan without incurring refractoriness whereas responses in the reversed direction require the creation of a new plan that is associated with refractoriness. In our joystick task, independent adjustments were only found in the sustained control conditions (i.e. in the unidirectional cases of the 1^st^ and 2^nd^ order systems). In these (velocity or acceleration controlled) conditions the properties of the system (inertia) make it unnecessary to select, plan, and initiate a second response in the same direction. Unlike the process of reversing direction, inhibition or attenuation of the ‘breaking action’ is sufficient to facilitate the ongoing movement in order to bring the system to its final (second) position.

### Interpretation of our evidence for refractoriness within the intermittent control framework

**Figure 7:**
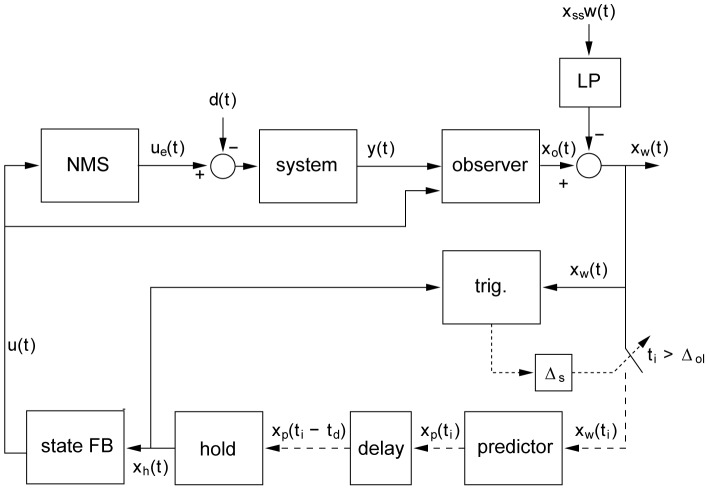
General model of intermittent control. The intermittent predictive controller is based on continuous control as a special case [12], [36], [43], but generally the predicted system state is only used intermittently to update the time varying control signal sent from the generalized “hold” to the actuator. “Trig.” detects when the control trajectory is to be updated and this event trigger requires three conditions: (i) a single event must be detected (i.e. all events within the sampling delay (Δs) are considered as one), (ii) a minimum open-loop interval (Δ_OL_) must have elapsed since the previous event and (iii) an error signal must exceed a threshold [12], [36], [43]. Scalar signals are represented by solid lines, vector signals are represented by dashed lines. The participant's neuro-muscular dynamics are modelled (linear) in the “NMS” block with input *u*(t). The linear external controlled system with output *y*(t) (represented by the “System” block) is driven by signals *u*_e_(t) and *d*(t) representing the externally observed control signal and the disturbance signal. The state of the composite “NMS” and “System” blocks is estimated *x_o_*(t) by the “observer” block. Sampling is preceded by an anti-aliasing low-pass filter “LP” of the subtracted set point disturbance *w*(t) and subject to an event delay “Δ_S_” between event and sampling. The trigger for the sampling times *t*_i_ is provided by the event detector block labelled “trig”. Sampling *x*_w_(t) takes place at discrete times ti. Sampled signals (represented by the dotted lines) are defined only at the sample instants *t*_i_. The future state error *x*_p_(ti) is provided by the “predictor” block. The various delays in the human controller are accounted for by a pure time delay of *t*_d_ represented by the “delay” block. The block labelled “hold” is a system-matched hold, that provides the delayed version of the continuous-time signal that is multiplied by the feedback gain vector *k* (block “State FB”) to give the feedback control signal *u*(t). This figure and its caption are based on [12].

**Figure 8:**
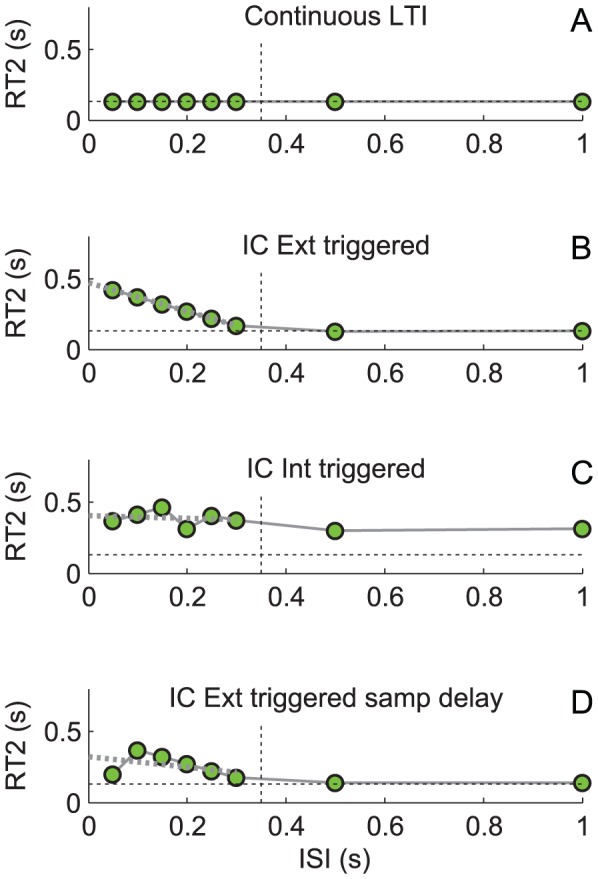
Model based interpretation (stage 3). Discriminating serial ballistic (intermittent) from continuous control and identifying several possible relationships between RT2 and inter-step interval (ISI). The simulated system is zero order. The open-loop interval (delta OL) is 0.35 s and feedback time delay (td) is 0.14 s. For four models: A) continuous LTI, B) externally-triggered intermittent control, C) internally-triggered intermittent control (triggered to saturation), and D) externally-trigger intermittent control supplemented with a sampling delay. The following is shown as a function of ISI: (A–D), mean RT2 (joined green circles), black dotted horizontal shows mean RT2 without interference, black dotted vertical shows the set open-loop interval, gray dotted shows unconstrained regression linear fit between RT2 and ISI using ISIs smaller than delta OL.

The intermittent control model's control signal (see diagram in Fig. 7.) is open loop for a minimum duration known as the intermittent open-loop interval and this feature discriminates continuous from intermittent control [13], [36]. Even though this model is an explicitly single channel hypothesis model, depending on parameter settings, there are several possible relationships between RT2 and ISI. Fig. 8 shows RT2 v ISI for a variety of intermittent control parameter settings [c.f. 36]. Consistent for all parameter settings, the intermittent interval is shown by the ISI up to which RT2 was delayed relative to RT1 (Fig. 8 B–D).

If the intermittent interval is zero, control is continuous and RT2 shows no change with ISI (Fig. 8, A). Our results (identification of refractoriness) reject this interpretation.

At low ISI, below the intermittent interval, the slope of the relationship between RT2 and ISI need not be exactly −1, even for this explicitly single channel model. When events are triggered externally at one event per step stimulus the slope is −1 (Fig. 8B). If additional events are triggered, by an internal error signal crossing a threshold (e.g. due to increased noise) the slope will be less than −1. Applying noise to the system or ultimately setting the event threshold to zero provides different examples of events being triggered internally at the maximal possible rate such that events are limited by the intermittent interval the slope will be −0.5 (Fig. 8 C). If noise is high enough [c.f. 36], the IC model does not define the relationship between RT1 and RT2 vs. ISI and any distinction between first and second response times breaks down and the slope is zero. Depending on parameter settings for noise levels and event thresholds, varying slopes between −1 and 0 can be simulated.

At the lowest ISI, RT2 need not increase as ISI decreases. Supplementing the intermittent control model with low pass filtering of the set-point and a sampling delay (i.e. the delay between the event and the sampling instant c.f. Fig. 7) leads to RT2 decreasing as ISI decreases leading to a peak in RT at a certain ISI, equal to the sampling delay (Fig. 8D). This feature, does not occur in previously published versions of the IC model [e.g. 12], [36], [43], but has been introduced to reproduce the amplitude transition function (ATF) effect observed by Barrett & Glencross [59], [60], in which participants combine their responses to first and second steps stimuli when ISIs are very small. The fact that an explicitly intermittent control model implementing a single channel hypothesis can produce alternative relationships between RT2 and ISI precludes unambiguous interpretation of the results.

We apply the following principles to interpret our results (stage 3 of our method of analysis). First we identify the open-loop interval from the ANOVA metric (i.e. the ISI up to which RT2 was significantly delayed relative to RT1, Fig. 6). Next, the refractory duration indicated by the intercept of the unconstrained regression slope (Fig. 6) allows us to infer: i) the degree to which events are fully triggered by external stimuli (Fig. 8, B) or, at the other side of the spectrum, internally triggered at a maximum rate determined by the minimum intermittent interval (Fig. 8, C), ii) the possibility that participants combining their responses to first and second steps stimuli when ISIs are very small (Fig. 8, D).

Using these principles we make the following deductions. Our best estimate of the open-loop interval (The ANOVA metric) increased with system order (c.f. Fig. 6) as in fact did all the other estimates of the refractory duration (i.e. the intercept of the unconstrained regression fit, the intercept of the −1 regression fit, and the Range in RT2). Thus we conclude that, regardless of the applied metric, the intermittent interval increased with system order. The slope of the unconstrained regression line decreased with system order. This indicates that events have a greater tendency to be triggered internally rather than by external stimuli with increasing system order. This interpretation supports the idea that in sustained control (i.e. 1^st^ and 2^nd^ order systems), event triggering is part of an ongoing control process and not just related to the external step stimuli.

Our results also show some evidence of a peak in RT2 that is particularly existent in the second order system conditions (c.f. Fig. 6, C and D) which may support the idea of a sampling delay. Participants seem to combine first and second responses for ISIs smaller than 250 ms which is indicative of a sampling delay somewhere between 150 and 250 ms. Our experiment was designed to minimize temporal and spatial predictability of the step stimuli. Since participants could not pre-program their responses, this implies that for ISIs larger than the sampling delay the second step incurred refractoriness.

### Why is the refractory duration so long?

One theory of intermittency [24], [25] relates high frequency clock-driven refractoriness with an intermittent interval of 50–100 ms to tremor and resonance related discontinuities at a frequency of 7–10 Hz. The intermittent intervals observed here of 250, 350, and 550 ms for zero, first and second order systems relate to control actions of two to four actions per second. This frequency of control is clearly different from the high frequency theory and falls most likely within the voluntary control bandwidth.

Do these low frequency responses reflect the hard physiological limits of the system or do they represent a preferred rate optimizing some soft criterion? If the intermittent interval is related to the feedback loop time delays, then we have to consider whether the relevant delays are the minimum transmission times within the neural circuitry, the time needed to process higher order (complex) input-output relations, or the time lags associated with evolution of state. Whereas the first relates to the intrinsic hard limits within human physiology, the latter two are related to the order of the external system that is being controlled. The fact that the refractory duration increases with system order implies that the intermittent interval is flexibly selected to be appropriate for the system rather than to be physiologically intrinsic. Humans are predisposed for a second order world in which systems follow Newtonian (2^nd^ order) dynamics. Through intermittency, we might have adopted a strategy to deal with the relatively large time delays involved in this kind of control making us more flexible and more resistant to perturbations.

### Applicability to other tasks

Our results and reasoning support the idea that refractoriness is associated with response planning and response selection within discrete and sustained movement control.

An open question is whether refractoriness applies generally to human movement control. It is important to realise that control which seems continuous might in fact be serial ballistic in nature (e.g. continuous joystick contact in [13] masqueraded tapping like behaviour). As discussed in [13] serial ballistic control is likely related to the bandwidth of voluntary control and thus would also apply to (normal) continuous contact control. Our current study supports that argument and strengthens the case that continuous contact manual control is serial ballistic in nature.

Are the mechanisms involved in rudimentary control like human posture and the mechanisms governing multi-segmental (voluntary) movements like human balance also serial ballistic in nature or does intermittency apply only to a subset of the human movement repertoire? If multi-segmental human balance involves a higher level of control compared to the more rudimental, peripheral, high-bandwidth (reflex) feedback mechanisms dedicated to maintaining individual joint angles of human posture, this would suggest that while both serial ballistic control and continuous control are universal control mechanisms; continuous mechanisms lie embedded within the more executive intermittent control mechanisms c.f. 22,^23^.

Part of the power of the current paper lies in the fact that we have focussed the experiment on the simplest possible test of the existence of refractoriness in sustained control. In particular, we have avoided model-selection issues by familiarising subjects before each trial and not changing the system during a trial and we have avoided multi-segmental issues [c.f. 61] by using a single-input, single output system. Having established the intermittent paradigm in this basic case, investigating model-selection and multi-segmental systems are challenges for the future.

### Conclusion

Following our recent demonstration that continuous control of second order systems is unnecessary [13], we asked whether refractoriness of substantial duration (∼200 ms) is evident in sustained contact control of external systems. We asked whether the refractory duration (i) is physiologically intrinsic, (ii) depends upon system order (zero, 1^st^, 2^nd^) or passive stabilisation (marginally stable, unstable) (iii) depends upon target jump direction (reversal, same direction). Thirteen participants used discrete movements (0 order external system) as well as more sustained control activity (1^st^ and 2^nd^ order external systems) to track unpredictable step-sequence targets. Results show a substantial refractory duration that depends upon system order (150–300, 200–500, 250–650 ms for 0, 1^st^ 2^nd^ order respectively, n = 13, p<0.05) but, in sustained control, only when the target reverses direction. We found no differences in results between the marginally stabilized and unstable second order systems. We propose that central refractoriness is an appropriate control mechanism for accommodating time varying systems, actuators, constraints including the more variable processing times of higher order (complex) input-output relations. Whether or not, intermittent mechanisms explain sustained control had been an open question for many years. While we cannot formally exclude alternative unmodelled explanations, our findings show that refractoriness is present in sustained control and can be best interpreted as intermittent rather than continuous control.
